# Patterns of Tobacco Smoking and Nicotine Vaping among University Students in the United Arab Emirates: A Cross-Sectional Study

**DOI:** 10.3390/ijerph18147652

**Published:** 2021-07-19

**Authors:** Luai A. Ahmed, Marina Verlinden, Mariam Ali Alobeidli, Reem Hamad Alahbabi, Radeya AlKatheeri, Basema Saddik, Abderrahim Oulhaj, Rami H. Al-Rifai

**Affiliations:** 1 Institute of Public Health, College of Medicine and Health Sciences, United Arab Emirates University, Al Ain P.O. Box 17666, United Arab Emirates; luai.ahmed@uaeu.ac.ae (L.A.A.); aoulhaj@uaeu.ac.ae (A.O.); 2 Department of Psychology, College of Natural and Health Sciences, Zayed University, Abu Dhabi P.O. Box 144534, United Arab Emirates; Maryna.Bondaruk@zu.ac.ae; 3 College of Medicine and Health Sciences, United Arab Emirates University, Al Ain P.O. Box 17666, United Arab Emirates; 201400959@uaeu.ac.ae (M.A.A.); 201412927@uaeu.ac.ae (R.H.A.); 201304934@uaeu.ac.ae (R.A.); 4 Department of Family and Community Medicine, College of Medicine, University of Sharjah, Sharjah P.O. Box 27272, United Arab Emirates; bsaddik@sharjah.ac.ae

**Keywords:** electronic nicotine delivery system, e-cigarettes, midwakh, smoking, United Arab Emirates

## Abstract

Various forms of tobacco smoking and nicotine vaping tools are available on the market. This study quantified the prevalence of and identified factors associated with patterns of smoking and nicotine vaping among university students in the United Arab Emirates (UAE). A cross-sectional sample of students enrolled in three public universities was surveyed. Self-reported current smoking and nicotine vaping were recorded. Of 1123 students, 81.7% completed the online survey (mean age, 20.7 ± 3.4 (SD) years; 70.7% females). The prevalence of current smoking was 15.1% while the prevalence of current nicotine vaping was nearly 4.0%. Among current smokers, 54.7% reported conventional smoking only, 15.1% reported nicotine vaping only, and 28.8% were poly-users. Conventional midwakh (47.5%), followed by conventional shisha/waterpipe (36.7%), conventional cigarettes (36.7%), electronic shisha/waterpipe (25.2%), and electronic cigarettes (24.5%), were most commonly reported by students. Students aged 20–25 years (adjusted odds ratios (aOR): 2.08, 95% confidence interval (CI): 1.18–3.67) or >25 years (aOR: 4.24, 95% CI: 1.41–12.80) had higher odds of being current smokers compared to those aged 17–19 years. The male gender was also independently associated with higher odds of being a current smoker (aOR: 5.45, 95% CI: 3.31–8.97) as well as higher odds of smoking cigarettes, shisha, and midwakh, or nicotine vaping compared to being female. Of nicotine vaping users, 36.1% reported using nicotine vaping because they enjoyed the flavor and vaporizing experience and 34.4% used it to help them to quit smoking. A relatively high prevalence of self-reported smoking was reported among university students in the UAE. The findings also suggest that nicotine vaping use is relatively widespread, but still less common than traditional smoking. Vigilant and tailored university-based smoking control and preventive measures are warranted.

## 1. Introduction

The World Health Organization (WHO) estimated that 20.2% of the world’s population aged ≥ 15 years were current smokers in 2015 [1]. This estimate indicated that smoking rates have decreased by 6.7% globally since 2000 [1]. The Eastern Mediterranean Region (EMR) is expected to achieve an 11% relative reduction in smoking rates by 2025; however, the projected reduction in the EMR is slow compared to other regions [1]. This may be due to the increasing use of not only cigarette smoking but also other forms of tobacco smoking, especially shisha (also known as waterpipe, hookah, or narghile) [2] and midwakh [3,4]. Shisha smoking was reported to be associated with significant adverse effects on health, including the cardiovascular and respiratory systems [5,6], in addition to the negative economic impact on health costs and the gross domestic product, for both individuals and society [7,8]. Midwakh smoking, in which a small pipe is used to smoke a blend of dried tobacco leaves mixed with spices, herbs, or barks, known as “dokha” [9], was reported to be associated with several adverse health outcomes including, but not limited to, seizures [10] and acute effects on systolic blood pressure, heart rate, and respiratory rate [11]. Data on nicotine vaping use, including electronic cigarettes (e-cigarettes) and other electronic nicotine delivery systems (ENDS) (hereafter termed “e-cigarettes/ENDS”), are not available on a country-specific level.

The use of ENDS and vaping as an alternative to tobacco smoking is emerging [12]. E-cigarettes/ENDS are not harmless, but they are substantially less harmful than conventional cigarettes [13,14]. Although the use of e-cigarettes was recommended as an aid to quit smoking [14], research regarding its effectiveness as a smoking cessation tool is inconclusive [15,16]. A recent meta-analysis did not reach conclusive evidence in this regard [17], and concluded that there is very low certainty of evidence supporting e-cigarettes to help quit smoking in the short term, while there was not enough evidence to determine if e-cigarettes are a safe and efficacious means of smoking cessation in the long term [17]. On the other hand, it remains unclear whether e-cigarettes/ENDS are an alternative to or a substitute for conventional forms of tobacco smoking.

In the UAE, one of the Gulf Cooperation Council (GCC) countries, the prevalence of smoking is relatively high and it is most frequently seen in younger men [4,18,19,20]. Recently, a study reported that the prevalence of current tobacco use was 36% among men and 3% among women based on self-report, while the prevalence was 42% among men and 9% among women based on biochemical verifications [21]. Moreover, as in other GCC countries, midwakh use is highly popular in the UAE [4,21,22]. However, there is a lack of data about e-cigarette/ENDS use in the region. Considering that poly-use is common in the region, it is likely that alternative combinations of tobacco smoking (i.e., e-cigarettes/ENDS) may be also common [23].

Given the lack of detailed surveillance data, the burden of various patterns of smoking (such as shisha and midwakh), e-cigarette/ENDS use, and smoking cessation among university students has not yet been assessed in the UAE. This study bridges this knowledge gap by (1) assessing the prevalence of and identifying sociodemographic and economic factors associated with various patterns of tobacco smoking and nicotine vaping, and smoking cessation among university students in the UAE, and (2) exploring reasons for using e-cigarettes/ENDS.

## 2. Materials and Methods

### 2.1. Study Design

This cross-sectional survey was conducted between December 2019 and February 2020 among students enrolled in three main public universities in the UAE: the UAE University in Al Ain, Zayed University in Abu Dhabi and Dubai, and the University of Sharjah in Sharjah. All students enrolled in the undergraduate and postgraduate academic programs were eligible to participate.

Assuming a confidence level corresponding to a 95% confidence interval (CI; z = 1.96), an expected smoking prevalence of 27.7% in the study population (as reported in a recent study of college students in Saudi Arabia [24]), and a precision level of d = 0.05, sample and power calculations indicated that the required minimum sample size for a cross-sectional survey was 308. Accounting for a possible 20% non-response rate, we aimed to survey at least 370 students.

In collaboration with the students’ affairs office, a mass email was sent to all students. Faculty members helped to promote the study. Students were also informed about the study objectives and anonymity of participation, and they were required to sign an electronic consent form to participate in the survey. Participation was voluntary, and the participants were able to opt out at any time. Data were collected anonymously using an online self-administered questionnaire.

### 2.2. Data Collection Instrument

A brief questionnaire was developed that contained questions about the sociodemographic characteristics of the students and their current and previous smoking status. The self-administered questionnaire was first developed in English and then translated into the Arabic language and back-translated into English. The survey was piloted and minor corrections and amendments were made. The survey was administered in a bilingual format (Arabic and English).

The first part of the online survey contained an introduction about the study objectives, study importance, expected outcomes, and methodology, followed by a request to provide informed consent for voluntary participation. The participants were informed that the survey was completely anonymous, and no personal identifiers would be collected. The second part of the survey contained questions (seven items) concerning sociodemographic information, including sex and age, as well as nationality, emirate of origin, average monthly household income, current academic year, and degree program enrolment. The third part of the online survey addressed tobacco use. The participants provided information about their current smoking status (current smoker or quitter). Current smokers/users were then asked about the product they used: conventional smoking, e-cigarettes, e-shisha/waterpipe, e-pipe (cigar), snuff/chewing, and midwakh smoking. The participants also reported the age at which they first started smoking and how often they smoked at present. Finally, the students who reported e-cigarette/ENDS use were asked why they used e-cigarettes/ENDS. Seven potential answers were adapted from the Global Adult Tobacco Survey [25]. 

### 2.3. Data Analyses

Results based on continuous variables were reported as means with standard deviations (SD). The proportion of current smokers/users was estimated from the total number of students. The proportion of students who used each pattern of smoking/vaping was estimated from the total number of students. A multivariate logistic regression model was used to explore the factors associated with current smoking compared with never smoking. Multivariate models were also used to explore the factors associated with cigarettes, shisha, midwakh, and e-cigarette/ENDS use compared with never smoking. The multivariate logistic regression models included the following covariates: age, sex, and income. The crude odds ratios (ORs) and adjusted odds ratios (aORs) and their 95% CIs were reported. In addition, we examined factors associated with smoking cessation. A *p*-value of <0.05 was considered statistically significant. Data analyses were performed using the IBM SPSS v.25 statistics software [26].

The findings are reported in accordance with the STROBE guidelines for reporting cross-sectional studies [27].

## 3. Results

**Figure 1:**
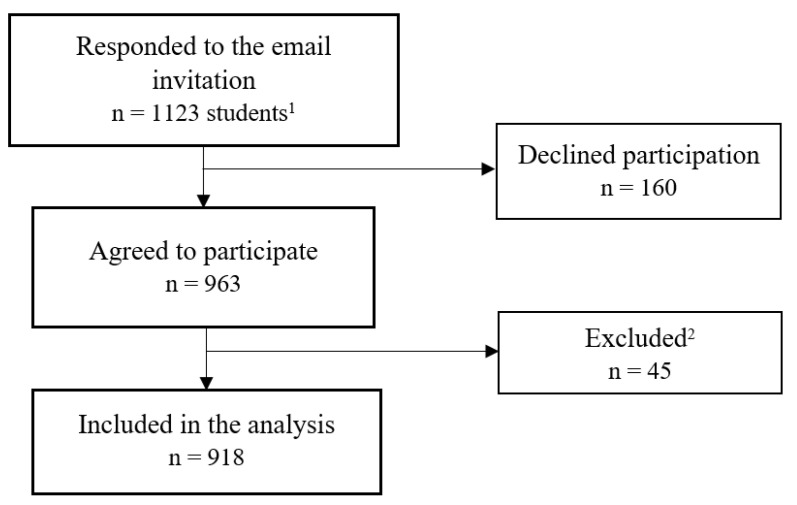
Flow chart of students’ responses from the three participating universities. ^1^ From the United Arab Emirates University, University of Sharjah, and Zayed University. ^2^ Reason for exclusion: consented but did not proceed with the survey (*n* = 41) and consented but did not report on their tobacco smoking status (*n* = 4).

A total of 1123 students accepted the email invitation, of whom 963 (85.8%) consented to participate and 918 (81.7%) were included in the analysis (Figure 1). The mean age of the students was 20.7 (SD = 3.4) years. Most of the students were female (70.7%) and Emirati (71.9%) and enrolled in the undergraduate program (93.8%). Current smoking or e-cigarette/ENDS use was reported by 139 (15.1%) students. Among the 918 students, conventional cigarettes (5.5%), conventional shisha (5.5%), and conventional midwakh (7.2%) were the most common patterns of tobacco smoking. E-cigarette and e-shisha/waterpipe use were reported by 3.7% and 3.8% of students, respectively (Table 1).

### 3.1. Patterns of Smoking and e-Cigarette/ENDS Use

Among tobacco smokers/nicotine vapers, the most frequently reported products (allowing for poly-use) were, in descending order, conventional midwakh (47.5%), conventional shisha (36.7%), conventional cigarettes (36.7%), e-shisha (25.2%), and e-cigarettes (24.5%). Of the current smokers/users, 54.5% were conventional tobacco users, 15.1% (21 out of 139) were e-cigarette/ENDS users, and 28.8% reported poly-use of both conventional and e-cigarettes/ENDS. Of the current smokers/users, over two-thirds (64.2%) had initiated smoking when they were 16–20 years old and 67.4% smoked on a daily basis. Current smokers/users differed significantly from non-smokers with regard to age (21.2 ± 3.5 vs. 20.6 ± 3.3 years, *p* = 0.039), sex (30.9% in males vs. 8.6% in females, *p* < 0.001), and household income (*p* < 0.001; Table 1).

Overall, 6.6% of the 918 students (43.9% of the current smokers/users) reported e-cigarette/ENDS use. E-cigarette/ENDS use was more common among those aged > 25 years compared to those aged 17–19 years (8.6% vs. 5.4%), males compared to females (10.1% vs. 5.3%), Emirati compared to Arab non-Emirati (7.0% vs. 5.3%), senior students compared to first-year students (7.1% vs. 5.2%), and students who initiated smoking before the age of <15 years compared to students who initiated smoking after the age of 20 years (22.2% vs. 10.5%). The two sexes were equally distributed among the 40 students who reported the dual use of conventional tobacco and e-cigarettes/ENDS (males and females, 20 vs. 20 students; Table 2).

Midwakh smokers and non-midwakh smokers differed with regard to sex (84.8% among males vs. 37.0% among females, *p* < 0.001), nationality (80.3% among Emirati vs. 57.5% among other nationalities, *p* < 0.05), marital status (98.5% among single/engaged vs. 87.7% among married, *p* < 0.05), and academic program (98.4% among undergraduates vs. 87.3% among postgraduates, *p* < 0.05) (Supplementary Table S1).

### 3.2. Factors Associated with Smoking and e-Cigarette/ENDS Use

Compared with students who never smoked, the multivariate logistic regression analyses showed that students aged 20–25 years (aOR: 2.08, 95% CI: 1.18–3.67) or >25 years (aOR: 4.24, 95% CI: 1.41–12.80) had higher odds of being current smokers than students aged 17–19 years. Students aged > 25 years had the highest odds ratio of being shisha smokers (aOR: 12.88, 95% CI: 3.31–50.01). Midwakh users were the only tobacco user group where the odds of use was higher among 20–25-year-olds compared to other age groups (aOR: 2.39, 95% CI: 1.01–5.62). Age was not associated with the use of e-cigarettes/ENDs; more likely, the number of observations was too low to reach the significance level (Table 3). Compared with females, male students had significantly higher odds of being current tobacco/e-cigarette/ENDS users (aOR: 5.45, 95% CI: 3.31–8.97) regardless of product type. The higher odds among males compared to females was especially apparent for midwakh users (Table 3).

Compared with a monthly household income of AED ≤ 14,999, students from a household with an income of AED ≥ 45K had higher odds of being current smokers (aOR: 2.15, 95% CI: 1.16–3.99). However, this significant association became insignificant (marginally insignificant with midwakh smokers, aOR: 2.41, 95% CI: 0.99–5.81) when smokers were categorized according to the exact pattern of smoking and the e-cigarette/ENDS use (Table 3).

Compared to non-midwakh smokers, the multivariate model revealed that male students had substantially higher odds of being midwakh smokers than females (aOR = 7.84, 95% CI: 2.93–21.00), while, compared to non-current smokers, being aged 20–25 years or a male student was associated with higher odds of midwakh smoking (aOR = 2.29, 95% CI: 1.03–5.13; aOR = 16.43, 95% CI: 7.07–38.21, respectively) (Supplementary Table S2).

### 3.3. Smoking Cessation

Of the 918 students, 12.6% reported having quit smoking. Smoking cessation was significantly more common among females and less affluent students (Supplementary Table S3). Males had lower odds of quitting smoking compared to females (aOR: 0.44, 95% CI: 0.23–0.82). Those who had quit smoking were more likely to have a medium household income (AED 15–44.9 K) compared with more affluent students with an average monthly income of AED ≥ 45 K (Supplementary Table S4).

### 3.4. Reasons for Using e-Cigarettes/ENDS

Of the 61 students who reported using e-cigarettes/ENDS, 34.4% and 36.1% reported using e-cigarettes/ENDS to help them quit smoking or because they enjoyed its flavor, respectively, whereas 24.6% and 21.3% believed that e-cigarette/ENDS use was less harmful than conventional tobacco, both to the users themselves and other people, respectively (Supplementary Table S5).

## 4. Discussion

This study revealed the prevalence of and identified sociodemographic factors associated with various patterns of smoking and e-cigarette/ENDS use among university students in the UAE. Overall, 15.1% of university students in the UAE were current smokers, of whom nearly 44.0% were e-cigarette/ENDS users. Midwakh was the most commonly used tobacco product. Older students were more likely to be current smokers, particularly shisha smokers, while male students were more likely to be cigarette, shisha, or midwakh smokers or e-cigarette/ENDs users than female students.

Compared to regional estimates, the prevalence of smoking (15.1%) among university students in the UAE in this study was lower than that reported among college students in Oman (23.5%) [28], undergraduate students in Syria (51.4%) [29], and male and female university students in Yemen (36.3% and 28.0%, respectively), Bahrain (27.0% vs. 4.2%, respectively), Tunisia (38.4% and 3.4%, respectively), Egypt (61.2% and 18.9%, respectively), Palestine (52.7% and 16.5%, respectively), and Jordan (54.3% and 11.1%, respectively) [30]. The prevalence of cigarettes (5.5%) and shisha (5.5%) smoking among university students in the UAE was far lower than that reported among UAE residents generally (37.7%) in 2018 [2], adolescents in Qatar (9.8%) [31], Libya (cigarettes: 80.2%) [32], Syria (cigarettes: 23.8%, shisha: 18.0%) [29], Jordan (cigarettes: 80.0%) [33], Saudi Arabia (cigarettes: 70.7%, shisha: 36.4–36.3%) [34,35], and Lebanon (shisha: 29.5%) [36], but slightly higher than the reported shisha smoking among university students in Bahrain (2.0%) and similar to the previously reported shisha smoking in Yemen (5.0%) [30] and among university students in Sharjah, UAE in 2005 (5.6%) [37]. In the UAE, enjoyment and friends’ influence were reported as common reasons for smoking shisha [2].

The observed prevalence of current midwakh smokers among university students in our study (7.2%) was lower than the prevalence of midwakh smoking reported among different population groups in the UAE (ranging from 12.5% to 39%) [4,11,18,19,20,38,39], but similar to the percentage of ever midwakh smokers among male students of Qassim University in Saudi Arabia (7.9%) and lower than that among current midwakh users (3.8%) [40]. Among tobacco smokers, the observed high rate of midwakh use (47.5%) in this study was similar to previous reports among secondary and high school students in the UAE [19,38,39]. The high rate of midwakh use (47.5%) by young adult smokers in our sample supports the need for addressing this public health problem. Such high use of midwakh in students draws attention to the need for early preventive measures since prior studies have shown that dokha (the tobacco used for midwakh smoking) is highly toxic [41,42]. Dokha has a higher concentration of nicotine and tar than other forms of tobacco [41], and it was also shown to have harmful effects on the oral microbiome and cardiovascular and respiratory systems [11,43]. The early initiation of midwakh use may continue into adolescence and young adulthood, increasing the risk of adverse health outcomes as a result of prolonged exposure to dokha. The potential popularity of midwakh use in the student population and young adults could be attributed to the less long-lasting smell, the ability to consume it discretely, and the fact that it requires a relatively small quantity of tobacco for each serving due to its high strength and concentration.

The prevalence of e-cigarette (3.7%) or e-cigarette/ENDS (~4.0%) use among university students in the UAE was slightly lower than what has been reported among medical students in two Saudi universities (7.2% [44] and 10.6% [45]), among school students in Canada (5.7%) [46], and among medical school students of the University of Minnesota (14.7%) [47]. The students believed that e-cigarettes/ENDS were less harmful to the user and other people. This observation is consistent with recent evidence from the UAE where 80.9% of e-cigarette users believed that e-cigarettes were less harmful than tobacco cigarettes [48]. This belief, and the increased popularity, might be influenced by the recent legalization of e-cigarettes in the UAE as these devices are commonly advertised and claimed by manufacturers to aid smoking cessation [48]. Overall, e-cigarettes/ENDS have gained a reputation of being less harmful and are more accepted by society [12], and they are reported to be effective as an aid for quitting smoking [14]. Moreover, the common use of e-cigarettes/ENDS by current smokers (43.9%, primarily in dual use with conventional tobacco) in this study may indicate the potential use of e-cigarettes/ENDS as an aid to quit smoking. Although the immediate or short-term effects of vaping appear to be less harmful than those of conventional smoking [25], the long-term effects have been inconsistently reported [13,14,49,50] or are not yet fully understood [43]. Health risks associated with e-cigarette/ENDS use could stem from nicotine dependence. Although nicotine itself is not a carcinogen [51], exposure to nicotine is associated with various long-term consequences [51,52].

Over the past few years, the e-cigarette/ENDS market has been less strictly regulated than the conventional tobacco one, and it relies substantially on Internet sales [53,54]. From this perspective, e-cigarettes/ENDS may be a major public health challenge because they are easily accessible, particularly by adolescents [55]. The e-cigarette/ENDS use among adolescents is high, and they appear to be popular [56]. They may serve as a precursor to later conventional smoking; additionally, the lack of smoke makes them popular among adolescents because it helps to elude detection by parents or teachers. Their popularity and use surged sharply in 2017–2018 and is likely to rise further in the coming years, which may be due to the higher public acceptance of this alternative form of smoking or its taste or cost [56,57,58]. More studies are necessary to monitor e-cigarette/ENDS use among university students.

Age, gender, and household income status were independently positively correlated with smoking. The finding of older students (≥20 years) being more likely to smoke compared to other age groups supports what has been previously documented [59,60,61]. This observed positive correlation with being older and more likely to smoke was more prominent with cigarettes and shisha smoking. The finding of a positive correlation between being male and a smoker regardless of the pattern of smoking, and with an observed stronger association with midwakh smoking, is also consistent with other comparable populations in the UAE [37] and other Arab countries, including Yemen [61], Palestine [62], Bahrain [63], Jordan [64], and Saudi Arabia [35,65,66]. In the literature, the higher prevalence of smoking among males has been attributed to the social acceptability of the smoking habit among males [67,68,69,70] as well as to the same sex peer influence in the smoking behavior [65]. Although students with higher household income status were more likely to be smokers compared to less well-off students, this significantly higher likelihood was not specifically associated with any particular product (cigarettes, shisha, midwakh, and e-cigarettes/ENDS). The positive association between smoking and income status might be attributed to the ability of students to financially tolerate the imposed excise taxation on smoking in the UAE [71]. On the other hand, the insignificant association of income status with each pattern of smoking might indicate equal diffusion of various patterns of smoking among university students regardless of their economic status. However, the diffusion of smoking and/or vaping among students from less advantaged social backgrounds is unlikely to indicate that the country is positioned in the late stages of the smoking epidemic [72]. This is justified by the observed significant gap in the prevalence of smoking among males compared to females. However, the observed prevalence of smoking and vaping among students from less advantaged social backgrounds might indicate, for example, that students are trying to use the more socially acceptable forms of smoking (midwakh and shisha) as well as that e-cigarettes/ENDS are used as an alternative to conventional forms of smoking or as an aid to help quit smoking. This finding might be influenced by the lack of awareness of smoking risks to health as well as the lack of conclusive evidence on the benefits and harms of smoking and vaping.

In 2008, the WHO introduced six MPOWER measures to help countries combat tobacco use through effective interventions that are proven to reduce the demand for tobacco [73]. Our results indicating that 12.6% of students quit smoking are very promising. This finding provides important and timely evidence that may reflect the potential impact of interventions being recently implemented in the UAE to reduce tobacco smoking. By 2021, the aim is to reduce tobacco consumption in the UAE to 15.7% among men and to 1.7% among women [74]. Moreover, the desire to quit tobacco smoking might be driven by the recently introduced 100% “excise tax” on tobacco products, including e-cigarettes/ENDS, across the country [71]. Other recently implemented tobacco control measures are: smoking in certain enclosed public spaces and public transport is prohibited, tobacco advertising is prohibited in all print and electronic media, and health warnings (pictorial and text) should cover no less than 50 percent of the bottom of tobacco product packaging’s main display areas. The law also forbids automatic vending machines and devices for tobacco distribution inside the country, and smoking in houses of worship, educational institutions (such as universities and schools), and health and sports facilities [74]. Additionally, in 2015, the Ministry of Health and Prevention in the UAE launched the first unified guidebook to help people quit smoking by implementing specialized clinics dedicated to helping people quit smoking, managed by staff with the necessary skills to effectively support individuals who are in the process of withdrawing from the use of tobacco [75]. Considering the high prevalence of smoking, particularly midwakh use, among the student population observed in this study, additional interventions and vigilant policies are warranted. Universities are a very convenient venue to reach a large proportion of the general population through raising students’ awareness via printed flyers and mass media campaigns using official channels (mass emails) about the harmfulness of smoking and the misconceptions of e-cigarettes/ENDS.

The observed large fraction of female students in the present study represents the actual gender distribution among university students in the UAE. Almost two-thirds of the university students in the UAE are female. However, we do not know the degree to which our results are generalizable to the entire population of university students and to the general population of a similar age in the UAE. Moreover, the cross-sectional design and possible selection bias limit the generalizability and ability to make causal inferences of our findings. Although our survey was completely anonymous, reporting bias cannot be ruled out. The self-reported smoking status could be underestimated, particularly among females, given sociocultural expectations. A recently published population-based study in the UAE reported that tobacco use in the UAE was found to be higher than previously believed, specifically among females [21]. It also demonstrated that although the self-reported smoking prevalence was 36% among men and 3% among women, biological samples showed that 42% of men and 9% of women were positive for cotinine [21]. The observed absence of a significant association between some measured exposures, although the estimate of association was high, may be due to insufficient statistical power given the limited sample size.

Despite these potential limitations, our study is one of the first to examine patterns of smoking and vaping, including e-cigarette/ENDS use, among university students in the UAE. The sample size was appropriate as only 370 participants would have been sufficient to detect a prevalence of 27.7%. Our results are highly relevant to researchers and public health professionals in the UAE and neighboring GCC countries, where the youth populations share similar sociocultural characteristics and where smoking rates and tobacco use patterns are likely to be very similar. Tobacco control efforts may require strengthening or imposing different strategies to prevent or mitigate smoking, particularly midwakh and e-cigarettes/ENDS. Further research, including systematic reviews and meta-analyses, is warranted to generate hypotheses about the level and burden of the various patterns of tobacco smoking and nicotine vaping among university students in the different countries in the region, and to discuss the role of related public policies and cultural factors.

## 5. Conclusions

A relatively high prevalence of self-reported smoking was reported among university students in the UAE, particularly the use of midwakh. The study findings also suggest that the prevalence of nicotine vaping (e-cigarettes/ENDS) is increasing, but it is still less common than traditional smoking. Vigilant and tailored university-based smoking control and preventive measures are warranted. Employment of electronic communication and social media channels to raise students’ awareness about the adverse health effects of smoking and to handle the poly-use of conventional forms of smoking and the emerging smoke vaping tools could contribute to ongoing control and preventive efforts.

## Figures and Tables

**Table 1:** Characteristics of participating students by their current smoking status (smoker, non-smoker).

Characteristics	All Students *n* (Column %)	Current Smokers/Users *n* (%) ^a^	Non-Smokers/Non-Users *n* (%) ^a^	*p*-Value
N (%)	918 (100)	139 (15.1)	779 (84.9)	
Age ^b^				
Mean	20.66 ± 3.38 SD	21.2 ± 3.5 SD	20.6 ± 3.3 SD	0.039
17–19 years	351 (38.2)	34 (9.7)	317 (90.3)	0.001
20–25 years	531 (57.9)	97 (18.3)	434 (81.7)	
>25 years	36 (3.9)	8 (22.2)	28 (77.8)	
Sex				<0.001
Male	269 (29.3)	83 (30.9)	186 (69.1)	
Female	649 (70.7)	56 (8.6)	593 (91.4)	
Nationality				0.559
Emirati	660 (71.9)	95 (14.4)	565 (85.6)	
Arab non-Emirati	206 (22.4)	36 (17.5)	170 (82.5)	
Other nationalities	52 (5.7)	8 (15.4)	44 (84.6)	
Household monthly income, AED				<0.001
≤14,999	152 (25.1)	29 (19.1)	123 (80.9)	
15,000–29,999	158 (26.1)	10 (6.3)	148 (93.7)	
30,000–44,999	133 (22.0)	17 (12.8)	116 (87.2)	
≥45,000	162 (26.8)	41 (25.3)	121 (74.7)	
*Missing*	*313*	*42*	*271*	
Marital status				0.645
Single/engaged	860 (93.7)	129 (15.0)	731 (85.0)	
Married ^c^	58 (6.3)	10 (17.2)	48 (82.8)	
Academic programme				0.519
Undergraduate	851 (93.8)	125 (14.7)	726 (85.3)	
Postgraduate	56 (6.2)	10 (17.9)	46 (82.1)	
Missing	11	4	7	
Academic year				0.174
1st year	209 (22.9)	22 (10.5)	187 (89.5)	
2nd year	303 (33.2)	50 (16.5)	253 (83.5)	
3rd year	209 (22.9)	37 (17.7)	172 (82.3)	
≥4th year	192 (21.0)	29 (15.1)	163 (84.9)	
Missing	5	1	4	
Current tobacco smoker	139 (15.1)			–
*Conventional cigarettes*	51 (5.5)	51 (36.7) ^d^	–	
*Conventional shisha*	51 (5.5)	51 (36.7) ^d^	–	
*Conventional midwakh*	66 (7.2)	66 (47.5) ^d^	–	
*Conventional pipe/cigar*	5 (0.5)	5 (3.6) ^d^	–	
*Conventional snuff/chewing*	3 (0.3)	3 (2.2) ^d^	–	
*Electronic cigarettes*	34 (3.7)	34 (24.5) ^d^	–	
*Electronic shisha*	35 (3.8)	35 (25.2) ^d^	–	
*Electronic pipe/cigar*	12 (1.3)	12 (8.6) ^d^	–	
Age at first smoke ^e^				
<15 years	–	18 (26.9)	–	–
16–20 years	–	43 (64.2)	–	
>20 years	–	6 (9.0)	–	
*Missing*	*–*	*72*		
Frequency of current smoking ^d^				
Daily	–	93 (67.4)	–	–
2–3 times a week	–	23 (16.7)	–	
>3 times a week	–	5 (3.6)	–	
A few times a month	–	17 (12.3)	–	
*Missing*		*1*		

AED: Emirati dirhams. SD: standard deviation. ^a^ Denominator is the total number of students within each subcategory (row %), otherwise indicated. ^b^ For the 49 students with missing information on age, age was replaced by the mean age. ^c^ Including seven divorced. ^d^ Denominator is the total number of current smokers. Percentages do not add up to 100 because of the dual/poly-use. ^e^ Valid percentages. Denominator is the number of current smokers.

**Table 2:** The distribution of single and dual of conventional smoking and e-cigarettes/ENDS use by the students’ sociodemographic and academic characteristics.

Characteristic	e-Cigarette/ENDS Use		e-Cigarettes/ENDS Only	Conventional Only	Dual Use of Conventional and e-Cigarettes/ENDS
	Yes	No			
N (%)	*n* = 61 (%)	*n* = 857 (%)	*n* = 21 (%) ^a^	*n* = 78 (%) ^a^	*n* = 40 (%) ^a^
Age	*p* = 0.469 ^b^		*p* = 0.392 ^c^		
17–19 years	19 (5.4)	332 (94.6)	7 (20.6)	15 (44.1)	12 (35.3)
20–25 years	39 (7.4)	490 (92.6)	14 (14.6)	57 (59.4)	25 (26.0)
>25 years	3 (8.6)	32 (91.4)	0 (0.0)	6 (66.7)	3 (33.3)
Missing		3			
Sex	*p* = 0.008 ^b^		*p* = 0.006 ^c^		
Male	27 (10.1)	241 (89.9)	14 (24.1)	24 (41.4)	20 (34.5)
Female	34 (5.3)	613 (94.7)	7 (8.6)	54 (66.7)	20 (24.7)
Missing		3			
Nationality	*p* = 0.674 ^b^		*p* = 0.186 ^c^		
Emirati	46 (7.0)	611 (93.0)	18 (18.8)	50 (52.1)	28 (29.1)
Arab non-Emirati	11 (5.3)	195 (94.7)	1 (2.9)	24 (68.6)	10 (28.6)
Other nationalities	4 (7.7)	48 (92.3)	2 (25.0)	4 (50.0)	2 (25.0)
Missing		3			
Household monthly income, AED	*p* = 0.291 ^b^		*p* = 0.311 ^c^		
≤14,999	10 (6.6)	142 (93.4)	3 (10.7)	18 (64.3)	7 (25.0)
15,000–29,999	9 (5.7)	149 (94.3)	3 (27.3)	2 (18.2)	6 (54.5)
30,000–44,999	8 (6.0)	125 (94.0)	3 (17.6)	9 (52.9)	5 (29.4)
≥45,000	17 (10.7)	142 (89.3)	7 (17.5)	23 (57.5)	10 (25.0)
Missing	17	299	5	25	12
Marital status	*p* = 0.484 ^b^		*p* = 0.694 ^c^		
Single/engaged	56 (6.5)	803 (93.5)	20 (15.5)	73 (56.6)	36 (27.9)
Married	5 (8.9)	51 (91.1)	1 (10.0)	5 (50.0)	4 (40.0)
Missing		3			
Academic programme	*p* = 0.090 ^b^		*p* = 0.318 ^c^		
Undergraduate	55 (6.5)	794 (93.5)	20 (16.1)	69 (55.6)	35 (28.2)
Postgraduate	3 (5.5)	52 (94.5)	0 (0.0)	8 (72.7)	3 27.3)
Missing	4	11			
Academic year	*p* = 0.333 ^b^		*p* = 0.826 ^c^		
1st year	11 (5.2)	199 (94.8)	4 (18.2)	11 (50.0)	7 (31.8)
≥2nd year	50 (7.1)	650 (92.9)	17 (14.7)	66 (56.9)	33 (28.4)
Missing		8			
Age at first smoking	*p* = 0.499 ^b^		*p* = 0.526 ^c^		
<15 years	10 (22.2)	35 (77.8)	3 (16.7)	8 (44.4)	7 (38.9)
16–20 years	18 (16.7)	90 (83.3)	8 (18.6)	25 (58.1)	10 (23.3)
>20 years	2 (10.5)	17 (89.5)	0 (0.0)	4 (66.7)	2 (33.3)
Missing	31		10	41	21

ENDS: electronic nicotine delivery systems. AED: Emirati dirhams. ^a^ Denominator is the number of current smokers in each subcategory (row %). ^b^ *p*-value assessing the difference in proportions among the total population using the Chi-square test. ^c^ *p*-value assessing the difference in proportions among the current smokers using the Chi-square test.

**Table 3:** Adjusted associations between sociodemographic and academic characteristics and different patterns of smoking e-cigarette/ENDS use.

Characteristic	Compared with Never Smoking				
	Smoker ^a^	Cigarette ^a^	Shisha ^a^	Midwakh ^a^	e-Cigarettes/ENDS ^a^
	aOR (95% CI)	aOR (95% CI)	aOR (95% CI)	aOR (95% CI)	aOR (95% CI)
Age					
17–19 years	1.00	1.00	1.00	1.00	1.00
20–25 years	2.08 (1.18–3.67) *	3.54 (1.31–9.58) *	2.53 (0.98–6.50)	2.39 (1.01–5.62) *	1.27 (0.63–2.53)
>25 years	4.24 (1.41–12.80) *	6.21 (1.26–30.56) *	12.88 (3.31–50.01) ***	1.38 (0.12–15.03)	2.62 (0.64–10.60)
Sex					
Female	1.00	1.00	1.00	1.00	1.00
Male	5.45 (3.31–8.97) ***	3.96 (1.89–8.28) ***	4.92 (2.32–10.40) ***	19.37 (8.10–46.31) ***	3.19 (1.64–6.21) **
Nationality					
Emirati	1.00	1.00	1.00	1.00	1.00
Arab non-Emirati	1.21 (0.69–2.14)	2.21 (1.00–4.89)	3.96 (1.79–8.78) **	0.58 (0.24–1.40)	0.58 (0.24–1.42)
Others	0.50 (0.16–1.54)	0.67 (0.13–3.56)	1.43 (0.32–6.36)	0.11 (0.01–1.01)	0.79 (0.20–3.08)
Household monthly income, AED					
≤14,999	1.00	1.00	1.00	1.00	1.00
15,000–29,999	0.38 (0.17–0.86) *	0.31 (0.08–1.18)	0.32 (0.08–1.21)	0.19 (0.04–0.91) *	0.88 (0.33–2.26)
30,000–44,999	0.93 (0.45–1.92)	1.26 (0.49–3.22)	1.40 (0.52–3.76)	1.29 (0.47–3.55)	0.97 (0.35–2.73)
≥45,000	2.15 (1.16–3.99) *	1.41 (0.56–3.56)	1.65 (0.66–4.20)	2.41 (0.99–5.81)	2.23 (0.94–5.27)
Marital status					
Single/engaged	1.00	1.00	1.00	1.00	1.00
Married	0.96 (0.32–2.91)	0.96 (0.22–4.26)	0.52 (0.09–2.97)	–	1.47 (0.42–5.09)
Academic programme					
Undergraduate	1.00	1.00	1.00	1.00	1.00
Postgraduate	1.28 (0.48–3.40)	1.51 (0.35–6.40)	3.74 (1.10–12.84) *	0.30 (0.02–4.85)	1.05 (0.22–4.95)
Academic year					
1st year	1.00	1.00	1.00	1.00	1.00
≥2nd year	1.33 (0.73–2.44)	5.25 (1.22–22.66) *	1.07 (0.46–2.49)	2.13 (0.79–5.74)	0.96 (0.46–2.00)

aOR: OR adjusted for age, sex, and income, except for academic year, which was not adjusted for age due to collinearity (covariates included as continuous variables). ENDS: electronic nicotine delivery systems. AED: Emirati dirhams. ^a^ Including those who reported the dual use of conventional and e-cigarette/ENDS excluding quitters. * *p* < 0.05, ** *p* = 0.001, *** *p* < 0.001.

## Data Availability

Upon reasonable justification, data could be made available to qualified researchers by request to the corresponding author.

## Supplementary Materials

The following are available online at https://www.mdpi.com/article/10.3390/ijerph18147652/s1, Table S1: Characteristics of participating students by their current midwakh smoking status. Table S2: Crude and adjusted characteristics associated with midwakh use compared with non-midwakh and non-current smokers. Table S3: Crude and adjusted characteristics associated with smoking cessation. Table S4: Characteristics of participating students by their current smoking status (current smoker vs. quitter and never smoker vs. quitter). Table S5: Reasons for using e-cigarettes/ENDS.
