# Supplementary material for: Patterns of Tobacco Smoking and Nicotine Vaping among University Students in the United Arab Emirates: A Cross-Sectional Study
Source: Int J Environ Res Public Health. 2021 Jul 19;18(14):7652. doi: 10.3390/ijerph18147652 (PMC8306162; doi:10.3390/ijerph18147652)
Supplement: Supplementary file 1 [file ijerph-18-07652-s001.zip › ijerph-1260331-supplementary/Supplementary Table S1.pdf]

Supplementary Table S1. Characteristics of participating students by their current midwakh smoking status

	**Current midwakh smoker** n = 66 (%)	**Non-current midwakh smoker** n = 73 (%)	P value[1]	**Non-current smoker** n = 779 (%)	P value[2]
Age			0.094		0.007
17–19 years	15 (22.7)	19 (26.0)		315 (40.4)	
20–25 years	50 (75.8)	47 (64.4)		435 (55.9)	
≥25 years	1 (1.5)	7 (9.6)		29 (3.7)	
Sex			<0.001		<0.001
Male	56 (84.8)	27 (37.0)		186 (23.8)	
Female	10 (15.2)	46 (63.0)		593 (76.2)	
Nationality			0.015		0.352
Emirati	53 (80.3)	42 (57.5)		564 (72.4)	
Arab non-Emirati	11 (16.7)	25 (34.2)		171 (22.0)	
Other nationalities	2 (3.0)	6 (8.2)		44 (5.7)	
Household monthly income, AED			0.395		0.001
≤14,999	13 (30.2)	16 (29.6)		124 (24.5)	
15,000–29,999	2 (4.7)	8 (14.8)		145 (28.6)	
30,000–44,999	9 (20.9)	8 (14.8)		117 (23.1)	
≥45,000	19 (44.2)	22 (40.7)		121 (23.9)	
Missing	*12*	*23*		*271*	
Marital status			0.014		0.114
Single/engaged	65 (98.5)	64 (87.7)		730 (93.7)	
Married[3]	1 (1.5)	9 (12.3)		49 (6.3)	
Academic program			0.014		0.134
Undergraduate	63 (98.4)	62 (87.3)		724 (93.9)	
Post-graduate	1 (1.6)	9 (12.7)		47 (6.1)	
Missing	2				
Academic year			0.271		0.032
1st year	8 (12.3)	14 (19.2)		186 (24.0)	
≥2nd year	57 (87.7)	59 (80.8)		589 (76.0)	
Missing	*1*			*4*	

AED: Emirati dirhams

[1] Midwakh vs. non-current midwakh of current smokers

[2] Midwakh vs. non-current smokers

[3] Seven were divorced
