# Supplementary material for: Patterns of Tobacco Smoking and Nicotine Vaping among University Students in the United Arab Emirates: A Cross-Sectional Study
Source: Int J Environ Res Public Health. 2021 Jul 19;18(14):7652. doi: 10.3390/ijerph18147652 (PMC8306162; doi:10.3390/ijerph18147652)
Supplement: Supplementary file 1 [file ijerph-18-07652-s001.zip › ijerph-1260331-supplementary/Supplementary Table S2.pdf]

Supplementary Table S2. Crude and adjusted characteristics associated with midwakh use compared with non-midwakh and non-current smokers

Characteristic	**midwakh vs. non-midwakh smokers**[1]		**midwakh vs. non-current smokers**[2]	
Age	OR (95% CI)	aOR (95% CI)	OR (95% CI)	aOR (95% CI)
17–19 years	1.00	1.00	1.00	1.00
20–25 years	1.34 (0.61–2.96)	1.17 (0.38–3.57)	2.41 (1.33–4.36)**	**2.29 (1.03–5.13)***
≥25 years	0.18 (0.02–1.64)	0.23 (0.02–2.67)	0.72 (0.09–5.66)	0.98 (0.10–9.40)
Sex				
Female	1.00	1.00	1.00	1.00
Male	9.54 (4.19–21.74)***	**7.84 (2.93–21.00)*****	17.95 (8.98–35.89)***	**16.43 (7.07–38.21)*****
Nationality				
Emirati	1.00	1.00	1.00	1.00
Arab non-Emirati	0.35 (0.15–0.79)*	0.37 (0.12–1.11)	0.68 (0.35–1.33)	0.55 (0.24–1.27)
Other nationalities	0.26 (0.05–1.37)	0.32 (0.02–1.15)	0.48 (0.11–2.05)	0.20 (0.03–1.63)
Household monthly income, AED				
≥45,000	1.00	1.00	1.00	1.00
30,000–44,999	1.30 (0.42–4.03)	0.78 (0.21–2.88)	0.49 (0.21–1.13)	0.58 (0.23–1.42)
15,000–29,999	0.29 (0.06–1.53)	0.16 (0.03–1.00)	0.09 (0.02–0.39)**	**0.09 (0.02–0.41)****
≤14,999	0.94 (0.36–2.45)	0.66 (0.21–2.05)	0.67 (0.32–1.41)	0.57 (0.25–1.29)
Marital status				
Single/engaged	1.00	1.00	1.00	1.00
Married (including seven divorced)	0.11 (0.01–0.90)*	–	0.23 (0.03–1.68)	–
Academic program				
Undergraduate	1.00	1.00	1.00	1.00
Post-graduate	0.11 (0.01–0.90)*	0.09 (0.00–1.62)	0.24 (0.03–1.80)	0.24 (0.01–4.27)
Academic year				
1st year	1.00	1.00	1.00	1.00
≥2nd year	1.70 (0.66–4.34)	2.03 (0.60–6.88)	2.25 (1.06–4.81)*	2.09 (0.82–5.32)
Age at first smoking			–	–
<15 years	1.00	1.00	–	–
16–20 years	0.63 (0.21–1.92)	0.45 (0.10–2.02)	–	–
>20 years	0.40 (0.06–2.77)	0.44 (0.04–5.58)	–	–

OR: odds ratio, aOR: OR adjusted for age, gender, and income, except for "Academic year", which was not adjusted for age due to collinearity (covariates included as continuous variables).

AED: Emirati dirhams.

[1] Including smokers only.

[2] Excluding other types of smoking.

* $p < 0.05$, ** $p = 0.001$, *** $p < 0.001$.
