# Supplementary material for: Patterns of Tobacco Smoking and Nicotine Vaping among University Students in the United Arab Emirates: A Cross-Sectional Study
Source: Int J Environ Res Public Health. 2021 Jul 19;18(14):7652. doi: 10.3390/ijerph18147652 (PMC8306162; doi:10.3390/ijerph18147652)
Supplement: Supplementary file 1 [file ijerph-18-07652-s001.zip › ijerph-1260331-supplementary/Supplementary Table S3.pdf]

Supplementary Table S3. Characteristics of participating students by their current smoking status (current smoker vs quitter and never-smoke vs quitter)

	**Quitter** n = 116 (%)	**Current smoker** n = 139 (%)	P value[1]	**Never-smoke** n = 663 (%)	P value[2]
Age			0.472		<0.001
17–19 years	36 (31.0)	34 (24.5)		281 (42.4)	
20–25 years	75 (64.7)	97 (69.8)		359 (54.1)	
≥25 years	5 (4.3)	8 (5.8)		23 (3.5)	
Sex			0.002		<0.001
Male	47 (40.5)	83 (59.7)		139 (21.0)	
Female	69 (59.5)	56 (40.3)		524 (79.0)	
Nationality			0.440		0.488
Emirati	84 (72.4)	95 (68.3)		481 (72.5)	
Arab non-Emirati	29 (25.0)	36 (25.9)		141 (21.3)	
Other nationalities	3 (2.6)	8 (5.8)		41 (6.2)	
Household monthly income, AED			0.040		<0.001
≤14,999	19 (24.7)	29 (29.9)		104 (24.1)	
15,000–29,999	20 (26.0)	10 (10.3)		128 (29.7)	
30,000–44,999	19 (24.7)	17 (17.5)		97 (22.5)	
≥45,000	19 (24.7)	41 (42.3)		102 (23.7)	
Missing	*19*	*42*		*232*	
Marital status			0.372		0.416
Single/engaged	104 (89.7)	129 (92.8)		627 (94.6)	
Married[3]	12 (10.3)	10 (7.2)		36 (5.4)	
Academic program			0.666		0.521
Undergraduate	109 (94.0)	125 (90.0)		617 (94.1)	
Post-graduate	7 (6.0)	10 (7.2)		39 (5.9)	
Missing		1 (0.7)			
Academic year			0.515		0.024
1st year	23 (19.8)	23 (16.5)		164 (24.9)	
≥2nd year	93 (80.2)	115 (82.7)		495 (75.1)	
Missing		1 (0.7)		4	

AED: Emirati dirhams

[1] Quitter vs. current smoker

[2] Quitter vs. never-smoker

[3] Seven were divorced
