# Supplementary material for: Patterns of Tobacco Smoking and Nicotine Vaping among University Students in the United Arab Emirates: A Cross-Sectional Study
Source: Int J Environ Res Public Health. 2021 Jul 19;18(14):7652. doi: 10.3390/ijerph18147652 (PMC8306162; doi:10.3390/ijerph18147652)
Supplement: Supplementary file 1 [file ijerph-18-07652-s001.zip › ijerph-1260331-supplementary/Supplementary Table S4.pdf]

Supplementary Table S4. Crude and adjusted characteristics associated with smoking cessation

Characteristic	Quitter vs. current smokers		Quitter vs. current non-smokers	
	OR (95% CI)	aOR (95% CI)	OR (95% CI)	aOR (95% CI)
Age				
17–19 years	1.00	1.00	1.00	1.00
20–25 years	0.73 (0.42–1.28)	0.57 (0.29–1.16)	1.63 (1.07–2.50)*	1.14 (0.68–1.92)
≥25 years	0.59 (0.18–1.98)	0.38 (0.09–1.54)	1.70 (0.61–4.79)	1.39 (0.434.46)
Sex				
Female	1.00	1.00	1.00	1.00
Male	0.46 (0.29–0.76)**	**0.44 (0.23–0.82)***	2.53 (1.67–3.83)***	**2.31 (1.38–3.85)****
Nationality				
Emirati	1.00	1.00	1.00	1.00
Arab non-Emirati	0.91 (0.52–1.61)	0.62 (0.29–1.30)	1.17 (0.74–1.86)	0.44 (1.52)
Other nationalities	0.42 (0.11–1.65)	0.18 (0.95–1.09)	0.42 (0.13–1.39)	0.13 (0.01–1.00)
Household monthly income, AED				
≥45,000	1.00	1.00	1.00	1.00
30,000–44,999	2.41 (1.03–5.65)*	**2.56 (1.07–6.12)***	1.05 (0.53–2.10)	1.09 (0.54–2.21)
15,000–29,999	4.32 (1.70–10.98)**	**4.33 (1.67–11.22)****	0.85 (0.43–1.67)	0.85 (0.43–1.69)
≤14,999	1.41 (0.64–3.13)	1.43 (0.63–2.25)	0.99 (0.49–1.98)	0.87 (0.43–1.78)
Marital status				
Single/engaged	1.00	1.00	1.00	1.00
Married	1.49 (0.62–3.58)	1.61 (0.55–4.71)	2.02 (1.02–4.0)*	1.49 (0.68–3.28)
Academic program				
Undergraduate	1.00	1.00	1.00	1.00
Post-graduate	0.99 (0.22–4.44)	0.50 (0.14–1.78)	1.02 (0.45–2.33)	0.70 (0.25–1.92)
Academic year				
1st year	1.00	1.00	1.00	1.00
≥2nd year	0.78 (0.40–1.46)	0.70 (0.29–1.70)	1.35 (0.83–2.19)	1.21 (0.64–2.28)
Age at first smoking				
<15 years	1.00	1.00	–	–
16–20 years	1.00 (0.50–2.05)	0.99 (0.41–2.38)	–	–
>20 years	1.44 (0.46–4.5)	1.30 (0.33–5.11)	–	–

AED: Emirati dirhams.

OR: odds ratio, aOR: OR adjusted for age, gender, and income, except for "Academic year", which was not adjusted for age due to collinearity (covariates included as continuous variables).

* $p < 0.05$, ** $p = 0.001$, *** $p < 0.001$.
