# Supplementary material for: Patterns of Tobacco Smoking and Nicotine Vaping among University Students in the United Arab Emirates: A Cross-Sectional Study
Source: Int J Environ Res Public Health. 2021 Jul 19;18(14):7652. doi: 10.3390/ijerph18147652 (PMC8306162; doi:10.3390/ijerph18147652)
Supplement: Supplementary file 1 [file ijerph-18-07652-s001.zip › ijerph-1260331-supplementary/Supplementary Table S5.pdf]

**Supplementary Table S5.** Reasons for using e-cigarettes/ENDS

| | N = 61 (%) |
|---|---|
| To help me quit tobacco smoking | **21 (34.4)** |
| Less harmless to me than conventional tobacco cigarettes | **15 (24.6)** |
| END has no nicotine | 7 (11.5) |
| To enjoy the flavour and vaporizing experience | **22 (36.1)** |
| Less harmless than conventional tobacco cigarettes to others | **13 (21.3)** |
| Culturally more acceptable than regular tobacco smoke | 11 (18.0) |
| Cheaper | 8 (13.1) |

ENDS: electronic nicotine delivery systems
